# Examining health literacy on cholera in an endemic community in Accra, Ghana: a cross-sectional study

**DOI:** 10.1186/s41182-019-0157-6

**Published:** 2019-05-08

**Authors:** Raymond Asare Tutu, Sangeeta Gupta, Janice Desire Busingye

**Affiliations:** 1 Global Societies Program, Delaware State University, Dover, DE 19901 USA; 2 Department of Public Health, Delaware State University, Dover, DE 19901 USA; 3 Kampala International University, P.O Box 20000, Kampala, Uganda

**Keywords:** Health literacy, Cholera, Tropical health, Accra slums, Ghana

## Abstract

**Background:**

The periodic and seasonal outbreaks of cholera in Ghana make the disease a vital health concern. The country is cholera endemic with several communities within cholera hotspots. This study, therefore, assesses health literacy on cholera and the association between health literacy competency and health outcome.

**Methods:**

The study adopted a health literacy framework that theorized the pathways between health literacy and health outcomes controlling for confounding factors. A survey questionnaire was administered to a representative sample of 401 individual household heads in James Town, Accra, Ghana. Reliability analysis was undertaken to ascertain the internal consistency of the instrument. Bivariate methods of analyses used were chi-square tests, ANOVA, Mann–Whitney *U* test, and Kruskal–Wallis test. Binary logistic regression models were run to examine the relative effects of health literacy competency on health outcome (having not had cholera).

**Results:**

There are substantial knowledge gaps about environmental risk factors for cholera like the presence of the cholera germ in coastal water, as well as the likelihood of contracting cholera due to overcrowded spaces. However, better knowledge on cholera risk factors was found to be associated with better health literacy competency (food safety and personal hygiene practices). An increase in health literacy competency score was associated with lower likelihood of having had cholera, after controlling for intermediate factors.

**Conclusion:**

Furthering health literacy on cholera environmental risk factors as well as a deliberate and targeted effort in encouraging consistency in the translation of disease knowledge into healthier practices may improve the well-being of the people.

## Background

### Introduction

Globally, cholera—a severe diarrheal disease caused by the consumption of food and water infested by the bacterium *Vibrio cholerae*—affects about 1.3 million to 4 million people annually and results in about 21,000 to 143,000 deaths [1]. The disease is geographically biased because some countries bear far more of the burden than others. In countries where cholera is endemic, an estimated 2.8 million cases of the disease are recorded annually and about 1.4 billion people are at risk [2]. There are several cholera hotspots identified in these endemic countries. Cholera hotspots are “specific and relatively small areas where the cholera burden is most concentrated and that play a central role in the spread of cholera” ([3] p. 7). The concentration of the disease in these hotspots fuels recurrent, periodic, and seasonal outbreaks. Consequently, mortality resulting from the disease in these places is high and it is compounded by the limited access to health care service [3, 4].

A lot of the hotspots are in Africa, and nearly 40 million to 80 million people live in cholera hotspots on the continent alone [3, 5]. The approximations of populations living in hotspot in some specific African countries include the Democratic Republic of Congo—23.8 million, Ethiopia—5.9 million, Nigeria—8.8 million, Kenya—2.8 million, Tanzania—6.5 million, and Cameroon—4.5 million [3]. In sub-Saharan Africa, from 2010 to 2016, except for Eritrea and Djibouti, an average of 141,918 cholera cases were reported annually with over 80 million people living in high cholera incidence geographic districts [6].

Ghana, like other countries in sub-Saharan Africa, has about 7.9 million of its people living in cholera hotspots [3]. The Ghana Health Service in 2016 reported a cholera outbreak which affected seven out of the ten administrative regions of the country [7], and the coastal regions are the high-risk areas experiencing increased occurrence and intensity of cholera epidemics [8]. The major risk factors identified for cholera epidemics in the country based on environmental assessment and research include open defecation practices, unsuitable dumping of refuse, safe drinking water inaccessibility, overflowing refuse dumps, blocked gutters, leaking sewage pipes, overcrowding in communities, poor personal hygiene, and poor food safety practices that result in food contamination [9–12]. Cholera outbreak in East Akin district of Ghana, which was waterborne, revealed the impact of environmental factors as evidenced by open defecation and sand-washing in the Birim river by artisanal miners, thereby exposing the community to the risk of cholera through sharing of contaminated water [9]. The relationship between poor sanitation, environmental hygiene, and cholera cases in the city of Accra has been explored [10, 11]. With respect to spatial distribution of the disease in the city, areas with poor sanitation due to inadequate sanitation services that are coupled with poor personal hygienic practices experience higher proportions of a cholera outbreak. For example, Oteng-Ababio [11] has demonstrated that during an outbreak of cholera in Accra, the rate of infection was 10 times higher in low-income communities where sanitation and environmental services (toilet facilities and garbage collection) are woefully inadequate or below par compared with the city average. Whereas 85% of the cases occurred in such low-income settlements, they are sometimes 100 times higher than the cases in more affluent parts of the city [11]. Substandard personal hygiene practices such as not washing hands frequently, not washing hands with soap, and untidy fingernails enhance the transmission of germs to human through food when consumed [10]. While Mensah et al. [10] found adequate frequency of personal hygiene behavior among their study population, food preparation process that required the use of hands repeatedly impacted food infection in Accra. Regarding food contamination, institutional food handlers are equally responsible. Although the major institutional food handlers in Ghana have “satisfactory knowledge” on food safety, knowledge does not always translate into standard hygienic attitudes and practices during food preparation [13]. For example, in a study of food safety knowledge, attitudes, and practices among public institutions in Ghana, Akabanda et al. [13] found that over 80% of their respondents (food handlers) had unsatisfactory attitudes regarding refreezing defrosted foods; this is also confirmed by Kunadu et al.’s [14] findings. Regarding food safety practices, over 80% of their respondents (food handlers) reported the lack of use of gloves during unpackaged food distribution, and about 60% did not wear aprons and masks when they ought to do so. These were suggested to be the major reasons for the high incidence of foodborne diseases in schools including cholera (also see [15, 16]). For example, in a study of the incidence of foodborne illnesses in senior secondary (high) schools in the Ashanti region of Ghana, Ababio et al. [17] found that, out of the 77% of their respondents who consume food provided by their schools, 52% had experienced foodborne illness about 3–12 times in an academic year.

The endemicity of cholera and other foodborne diseases is clearly illustrated for Ghana. While a couple of studies have investigated functional, interactive, and critical health literacy on foodborne diseases, as well as the development of a cholera-focused health literacy tool [18, 19], we could not find studies on the knowledge, attitudes, and practices of consumers in poor urban neighborhoods on cholera from a health literacy perspective. Therefore, this study attempts to examine individual’s basic knowledge of cholera risk factors, knowledge of cholera signs, personal hygiene practices, and food safety practices using a health literacy framework in a cholera-endemic community. Specifically, the study asks the following questions: (1) Are there associations between individual’s demographic characteristics and health literacy—knowledge and practices on cholera risk factors? (2) Is better basic knowledge on cholera risk factors associated with better health literacy competency (food safety and personal hygiene practices)? (3) Is health literacy competency significantly associated with health outcome (whether an individual reported to have had cholera or not in the last 6 months preceding the survey)?

### Conceptual framework: the health literacy and health outcome nexus

**Fig. 1:**
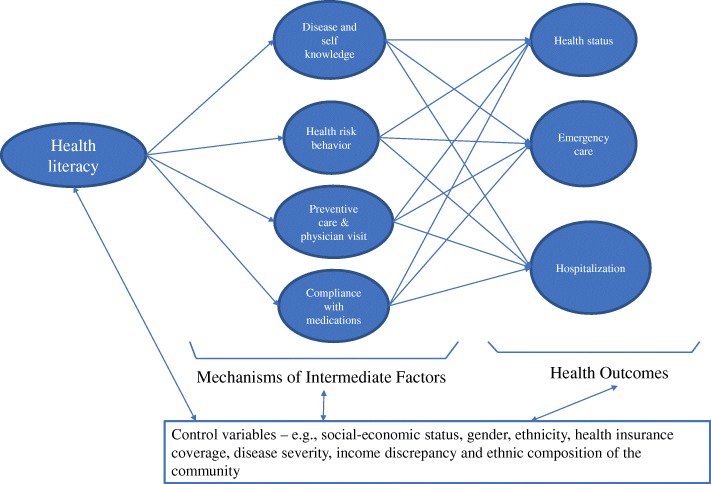
A conceptual framework for health literacy. Source: adapted from Lee at al. [32]

The concept of health literacy is over three decades old [20], and its growing importance in understanding health concerns is ubiquitous and cannot be overemphasized [21–24]. It has been comprehensively defined as “linked to literacy and entails people’s knowledge, motivation and competences to access, understand, appraise, and apply health information in order to make judgments and take decisions in everyday life concerning healthcare, disease prevention and health promotion to maintain or improve quality of life during the life course” ([25] p. 3). This all-embracing definition illustrates the multifaceted nature of health literacy and the plethora of dimensions that are associated with it. Among other things, it encompasses modules that illuminate our understanding on disease disparities, preventive care, measurement of individual capabilities and skills, healthcare utilization, and comprehension of prescription medication [26–31]. One of such health literacy models by Lee et al. [32] specifies four dimensions of relevance to health status. The health literacy framework (Fig. 1) integrates four mechanisms linking health literacy to three health outcomes (health status, emergency care, and hospitalization). These mechanisms (also referred to as intermediate factors) are knowledge of disease and self-care, health risk behavior, preventive care, and regular physician visits, as well as medication compliance. The model intimates that individuals with lower health literacy are likely to have unhealthier behavior, poorer regular preventive care, less medical knowledge, and shoddier medication compliance. Consequently, these factors may result in poor health outcomes such as increased hospitalization and emergency care utilization. These intermediate variables are mediated by individual socio-demographic confounders such as age, sex, income, education, ethnicity, and health insurance. Therefore, the model portrays the net effects of health literacy and the intermediate variables. The justification for the intermediate variables and their pathways to influencing health outcomes are as follows. Regarding disease knowledge and self-care, Lee et al. [32] draw on existing research that has unswervingly revealed that individuals with less health literacy have lower disease knowledge [33, 34]. With respect to health risk behavior, while they speculated that individuals with lower health literacy are more likely to be involved in risky health behavior like smoking and substance abuse, contemporary research has confirmed their assertion among varied populations [35, 36]. Concerning preventive care, it is suggested that inability to understand information about the significance of and need for early disease uncovering may result in lower utilization of preventive care. Therefore, concerns of functional literacy such as comprehension of physician instructions and directions may reduce individual’s access to preventive care. Pertaining to compliance with medication, factors leading to better compliance with medication are related to better functional and communicative health literacy. Research has shown that individual’s ability to interact effectively with the physician, ability to read labels, and comprehension of the doctor’s instructions are related to better medication compliance [33].

This study draws on two of the intermediate factors—disease knowledge and health risk behavior—to develop questions pertaining to knowledge, competence, and skills on cholera risk factors and prevention. Specifically, basic knowledge of cholera risk factors, knowledge of cholera signs, personal hygiene practices on cholera prevention, and food safety practices on cholera prevention are examined.

## Methods

### Study area

The study area is James Town. It is a neighborhood under the Ashiedu Keteke sub-Metropolitan Assembly in the Accra Metropolitan Area. Due to it exogenous characteristics (both physical and spatial) such as high population density, low housing standards, poor access to basic services and infrastructure including poor garbage disposal and inadequate toilet facilities, and being liable to flooding, it has been christened a matured slum by the Accra Metropolitan Assembly [37]. The lack of sufficient places of convenience, poor rubbish dumping, and inadequate private sources of pipe-borne water in the community are enough environmental risks to health and make residents vulnerable to diseases including foodborne ones [38]. Overcrowding, high population density, and large household sizes in restrictive spaces present unique circumstances for environmental risks to health in urban slums compared to rural communities. Meanwhile, for example, while open defecation in rural settings may be due to habits and preferences, in the urban slums, they are usually a result of insufficient toilet facilities, and these pose a public health danger to residents. It is, therefore, not surprising that the neighborhood is known to be cholera endemic and frequently reports other foodborne diseases such as dysentery and diarrhea [39].

### Sample and research instrument

The exponential population projection method was used to estimate the current population of James Town. Given a population of 15,508 as at the year 2010, at a growth rate of 2.2% and *t*-7, the current estimated total population of the community was 18,089. Using Epi Info software, a sample size of 377 was determined given the following power calculation statistics: confidence level = 95%, confidence interval (limit) = 5%, expected frequency (effect size) = 50%, design effect = 1, and cluster = 1. Although 10% more was added to the sample, a total of 401 individuals participated in this study. Research participants were aged 18 and above. James Town has been divided into 24 enumeration areas (census tracks) by the Ghana Statistical Service to facilitate the administration of censuses and surveys. Having undertaken a household listing task, participant households were systematically sampled from each of the enumeration areas.

A survey questionnaire was used for this study. It was divided into subsections. The first section constituted questions on the socio-demographic characteristics of the respondents including sex, age, education level, household size, income, marital status, and employment status. A section with 20 statement items measured on a Likert scale (strongly disagree—1, disagree—2, somehow disagree—3, agree—4, and strongly agree—5) elicited information on basic knowledge of cholera risk factors, knowledge of cholera signs, personal hygiene practices on cholera prevention, and food safety practices on cholera prevention. Respondents were asked if they had been told by a doctor that they have cholera in the last 6 months preceding the survey (yes—0, no—1). We chose the last 6 months preceding the survey for a couple of reasons. First, 6 months reduces the possibility of content errors due to memory lapse, especially among a population experiencing multiple diseases in the course of a year. Secondly, climatically, 6 months spans the wet and dry seasons, thus giving us the opportunity to capture some respondents who have had cholera. The questionnaire was administered (face-to-face interview mode) by 13 trained undergraduate research assistants in three main languages spoken in the area: English, Ga, and Twi. Each participant was interviewed in the language in which they were most comfortable. Each question was translated from English into Ga and Twi by professional and native speakers, and the training ensured consistency of intended meaning to all participants and, therefore, fostering high data quality. Wording of each statement on the questionnaire was reviewed repeatedly to avoid ambiguity and meanings lost in translation.

### Analyses of data

The methods of analyses adopted for this data were descriptive statistics, reliability analyses, chi-square tests, Mann–Whitney test, Kruskal–Wallis test, analysis of variance (ANOVA), correlation, and binary logit regression. Individual demographic characteristics are described followed by computation of Cronbach’s alpha coefficients to examine the internal consistency of the 20 statement items scale. Health literacy competency scores were computed using the nine statements on food safety practices and personal hygiene practices (see Table 1). Each respondent’s total score was the statistical sum of their scores per each item measuring food safety practices and personal hygiene practices, which were all measured on a Likert scale (strongly disagree—1, disagree—2, somehow disagree—3, agree—4, and strongly agree—5). That is, the coding was consistent across the nine items and all items were positively worded so there was no need for reverse coding. Thus, each specific item is assumed to have the same contributory weighting to the overall score. The relationship between basic knowledge of cholera risk factors and signs, demographic characteristics, and health literacy competency scores is examined using the ANOVA, Pearson correlation, Mann–Whitney test, Kruskal–Wallis test, and binary logistic regression.

**Table 1:** Percentage response distribution and mean scores on health literacy measures

Items	Mean (SD)	%, agreed/strongly agreed
Basic knowledge on cholera risk factors		
I know that I can get cholera through water that is contaminated	4.60 (0.18)	94.0
It is possible that I can gcet cholera through food	4.60 (0.81)	93.5
Practicing open defecation may be a way to spread cholera to me and my household	4.45 (0.84)	93.3
Cholera is caused by a germ	4.57 (0.79)	89.5
Personal hygiene practices		
I do wash my hands regularly because I do not want to get cholera	4.29 (1.08)	89.5
I always wash my hands with soap after I have used the toilet to avoid getting cholera	4.27 (1.00)	92.0
I often wash my body with soap daily to avoid getting cholera	4.60 (0.86)	85.0
I always wash my hands with soap before eating to avoid getting cholera	3.85 (1.32)	82.3
Knowledge on cholera environmental risk factors		
Crowded rooms and places may be a way that cholera can spread	4.11 (1.10)	66.1
During flooding situations, cholera can spread in the community and my household members and I can get cholera	3.81 (1.30)	79.1
In the event of water shortage, it is possible for cholera cases to increase in the community	4.49 (0.89)	68.1
The germ that causes cholera can be found in coastal water	3.44 (1.41)	55.6
Basic knowledge on cholera sign		
One sign that someone may have cholera is when he or she has profuse watery diarrhea	4.26 (1.04)	83.8
One sign that someone may have cholera is when he or she is vomiting profusely	4.15 (1.15)	79.1
One sign that someone may have cholera is when he or she is having leg cramps	2.84 (1.44)	32.7
Food safety practices		
My food is prepared in a clean environment in my household	4.39 (0.85)	87.8
I or whoever prepares my food washes their hands after they have used the toilet before preparing the food or touching water	4.29 (0.99)	80.0
The food I eat is always served hot	4.28 (1.03)	77.8
I always cover my food to prevent it from flies and other insects	4.45 (0.88)	88.3
I always store my cooking utensils in a clean and dry place	4.56 (0.70)	95.0

### Reliability, bivariate, and multivariate analyses

Reliability analyses were conducted to assess the internal consistency of the scale measuring health literacy competency on cholera. A satisfactory Cronbach’s alpha value of 0.711 was achieved for the scale of 20 items. At the bivariate analysis stage, chi-square tests were used to assess the relationship between demographic characteristics and specific individual items measuring basic knowledge of cholera risk factors, food safety practices, and personal hygiene practices. For significant relationships, Mann–Whitney test and Kruskal–Wallis test were used to ascertain the precise patterns of association. Further, one-way ANOVA was used to assess the relationship between health literacy competency score and basic knowledge of cholera risk factors. For this analysis, bearing in mind that all items were measured on the Likert scale (strongly disagree—1, disagree—2, somehow disagree—3, agree—4, and strongly agree—5), to make a clear distinction between those with better knowledge and those with poor knowledge, the basic knowledge of cholera risk factor responses were dichotomized. That is, responses from strongly disagree—1 to somehow disagree—3 were categorized as “disagree” while agree and strongly agree responses were classified as “agree.” Binary logistic regression models were run to investigate whether or not having had cholera in the last 6 months (health outcome) is associated with health literacy competency. A main effect term (model 1) with health literacy competency score and age (the independent variable with significant bivariate association with health outcome given an alpha value of 0.05) was built; then, an interaction term with both significant independent variables (model 2) was built; and finally, due to the conceptual framework, a main effect term with health literacy competency score and all the other demographic characteristics (model 3) was built. The model diagnostic results were favorable except for model 3. Specifically, the Hosmer and Lemeshow and variables in equation statistics indicated that the data fit the models well except for model 3 which had the Hosmer and Lemeshow test indicating statistical significance. For example, from the contingency table for the Hosmer and Lemeshow test for model 1, at the 10th step, given 41 observed number of respondents who did not have cholera in a group of subjects, the model predicted 40 of those respondents not having had cholera. The Hosmer and Lemeshow statistic assesses whether the observed event rates match that of the expected event rates in sub-groups of the population being modeled. Thus, “the advantage of the Hosmer–Lemeshow type tests is that they are based on groupings of the estimated probabilities that are intuitively appealing and easily understood by subject matter scientists” ([40] p. 968).

## Results

### Participant’s characteristics and health literacy measures

Forty percent of the research participants were male, and about 40% have had senior high school education or higher. About 74% were gainfully employed, whereas 68% identified with the Christian faith. Five percent reported having been told by a doctor to have had cholera within 6 months prior to the survey. Basic knowledge about cholera risk factors was almost universal. From Table 1, over 90% agree or strongly agreed that cholera, which is caused by a germ, can be contracted from contaminated water and food and practicing open defecation. However, there seems to be a considerable knowledge gap on other environmental risk factors. Over 40% of respondents disagreed that the germ that causes cholera can be found in coastal waters and that overcrowded places may be a way that cholera can spread. Concerning personal hygiene practices, while about 17% do not wash their hands with soap before eating, 8% do not always wash their hands after use of the lavatory. Regarding food safety practices, about 88% of respondents agreed or strongly agreed that their food is prepared in a clean environment whereas 12% reported that they do not always cover their food to prevent it from flies and other insects. Twenty-two percent disagreed that the food they eat is always served hot. Although over 80% knew that vomiting profusely and copious water diarrhea may be signs of cholera, only 33% knew that having leg cramps could be a cholera sign.

### Demographic characteristics, health literacy, and health outcome

Results from chi-square tests and ANOVA assessing the relationship between demographic variables and individual health literacy measures only showed significant associations between the following: (1) sex and two constructs of basic knowledge of cholera risk factors namely getting cholera through water and knowledge of getting cholera though food; (2) sex and one food safety practices measure, that is, covering food to prevent it from flies and other insects; (3) education level and one basic knowledge of cholera risk factor measure, that is, getting cholera through water. Results from Pearson correlation examining the association between demographic factors (age, income, and household size) and health literacy competency score did not yield any significant relationships.

From Table 2, we observed a statistically significant difference between the sexes. Mann–Whitney *U* tests reveal that females had a significant higher score than males on basic knowledge on cholera risk factors. Kruskal–Wallis test showed that those with junior high school education and above scored higher on knowledge of getting cholera through contaminated water compared to those with no or primary education. Each of the basic knowledge of cholera risk factors was significantly associated with health literacy competency score. Respondents who agreed that cholera can be contracted from contaminated water, food, and the practice of open defecation have a higher mean score on health literacy competency (positive food and hygiene practices) than their counterparts who disagreed (Table 3).

**Table 2:** Bivariate analysis of health literacy measures and demographic variables

Items	Sex	*N*	Mean rank
I know that I can get cholera through water that is contaminated^a^	Female	242	209.2
	Male	159	188.5
	Total	401	
It is possible that I can get cholera through food^b^	Female	242	208.6
	Male	159	189.4
	Total	401	
I always cover my food to prevent it from flies and other insects^c^	Female	242	204.1
	Male	159	196.2
	Total	401	
	Education level		
I know that I can get cholera through water that is contaminated^d^	No education	38	192.9
	Primary	49	190.8
	Junior high	152	206.0
	Senior high	132	206.3
	Tertiary	30	208.0
	Total	401	

Test statistics: ^a^[*Z* = − 2.357, *p* = 0.018]; ^b^[*Z* = − 2.075, *p* = 0.038]; ^c^[*Z* = 0.0775, *p* = 0.438]; ^d^[KWH 8.586, *p* = 0.0427]

**Table 3:** Bivariate analyses (ANOVA) of basic knowledge of cholera risk factors and health literacy competency score (food and hygiene practices)

Knowledge of cholera risk factors items		*N*	Mean	Std. deviation	*p* value
I know that I can get cholera through water that is contaminated	Disagree	24	33.6	4.3	0.035
	Agree	377	35.4	3.9	
	Total	401	35.3	3.9	
It is possible that I can get cholera through food	Disagree	26	33.4	4.0	0.011
	Agree	375	35.4	3.9	
	Total	401	35.3	3.9	
Practicing open defecation may be a way to spread cholera to me and my household	Disagree	27	33.8	5.5	0.042
	Agree	374	35.4	3.7	
	Total	401	35.3	3.9	
Cholera is caused by a germ	Disagree	42	33.5	5.0	0.003
	Agree	359	35.5	3.7	
	Total	401	35.3	3.9	

In model 1 (see Table 4), health literacy competency (food safety and personal hygiene practices) (Exp (*B*) = 1.109; *p* = 0.050) and age (Exp (*B*) = 1.041; *p* = 0.039) were significantly associated with the health outcome (having not had cholera in the last 6 months preceding the survey). In model 2, we found significant interaction terms between health literacy competency score and age. Specifically, the interaction effects indicated an Exp (*B*) of 1.001 (*p* = 0.015). The final model, which controlled for the other intermediate variables, did not show a significant relationship except for health literacy competency.

**Table 4:** Binary logistic regression of having had cholera six months preceding the survey

Variables	Categories	B	Model 1			Model 2			Model 3	
			S.E.	Exp(B)	B	S.E.	Exp(B)	B	S.E.	Exp(B)
Health literacy competency		0.103*	0.053	1.109				0.131*	0.059	1.140
Age		0.040*	0.019	1.041				0.036	0.023	1.036
Health literacy competency by Age					0.001*	0.001	1.001			
Educational level	No education (ref)									
	Primary							0.960	1.307	2.612
	Junior high school							1.769	1.315	5.868
	Senior high school							0.236	0.851	1.266
	Tertiary							1.138	0.932	3.119
Marital status	Single (ref)									
	Consensual union							−0.029	0.630	0.971
	Divorced							−1.385	0.816	0.250
	Separated							0.131	1.181	1.140
	Married							18.555	76.500	11.430
Employment status	Unemployed (ref)									
	Employed							−0.441	0.529	0.643
Religious affiliation	No religion (ref)									
	Christian							−17.056	23,059.109	0.999
	Muslim							−18.058	23,059.109	0.999
	Traditionalist							− 19.642	23,059.109	0.999

*p < 0.0

## Discussion

This study set out to investigate individual’s basic knowledge of cholera risk factors, knowledge of cholera signs, personal hygiene practices, and food safety practices using a health literacy framework in a cholera-endemic community. It is found that although knowledge about basic cholera risk factors such as contaminated water and food is almost unanimous in James Town, there seem to be considerable knowledge gaps about environmental risk factors for cholera such as the presence of the cholera germ in coastal water, as well as the possibility of contracting cholera due to overcrowded spaces. Demographically, females were found to have scored higher on basic knowledge on cholera risk factors, and respondents with higher education scored higher on basic knowledge on cholera risk factors. Additionally, better knowledge on cholera risk factors was found to be associated with better food and hygiene practices. An increase in health literacy competency score (food safety and personal hygiene practices) was associated with lower likelihood of having had cholera, before and after controlling for confounding factors. Increasing age was found to be associated with lower likelihood of having had cholera.

Whereas many individuals knew about the cholera infection through water and food, the finding about lower literacy on environmental risk factors for cholera in an endemic area in a coastal community is a cause for public health concern, especially given that environmental risk to health is pervasive in the neighborhood [37] and has been a major reason for susceptibility to foodborne diseases [9–11]. Therefore, being a coastal community, low health literacy on the presence of the cholera germ in coastal water is indicative of relative low disease knowledge, which has been argued to have the possibility of resulting in negative health outcome [33, 34].

Contrary to Akanbanda et al.’s [13] findings that knowledge about food safety practice did not translate into better food safety practices during preparation, our findings among individuals seem to suggest the opposite. Individuals with better knowledge on cholera risk factors had better food safety and personal hygiene practices such as covering food to avoid flies and other insects, washing of hands after the use of the lavatory, eating food that is served hot, and washing hands with soap. This finding is encouraging given that some public food handlers in Ghana, usually trained and certified, have been found not to translate their knowledge on food handling into attitude and practice comprising washing of hands, as well as using gloves and aprons [14].

More importantly, the seeming conversion of knowledge into practice is the very dream of health literacy practitioners and researchers, a situation where people’s knowledge enables access to health information thereby aiding in day-by-day personal decisions regarding disease prevention [25]. Consequently, the positive direct relationship between education level and knowledge on cholera risk factors was expected. The higher one’s education, the more likely it is for the person to know that cholera could be contracted through contaminated water. This is in consonance with research that has shown that knowledge of health risk factors, functional health literacy, and health literacy in general vary directly with education level [18, 19].

People with higher health literacy competency (better food safety and personal hygiene practices) were less likely to have had cholera. Healthier practices like preparation of food in a clean environment, frequently washing hands with soap, and storing cooking utensils in dry and neat places, despite the generally poor environmental conditions in the neighborhood, seem to have buffered against cholera infection. As Lee et al. [32] indicated, lower health literacy may result in unhealthier behavior with adverse consequences for health outcome. In this case, healthier behavior seems to have resulted in a better likelihood of not contracting cholera; this is in consonance with other studies that have looked at the link between behavior and health outcome [35, 36].

### Limitations

This present study has a couple of limitations worth highlighting. First, although the analyses showed a reliable instrument through an excellent internal consistency measure result, per Cronbach’s alpha value, the structure of the questionnaire has a technical flaw. Specifically, while the fourth item measuring knowledge of cholera risk factor seeks to examine whether respondents know that cholera is caused by a germ, a subsequent question asks whether the cholera germ is found in coastal water. Secondly, the use of only quantitative measures for health literacy knowledge and competency does not reveal the expanse of the knowledge of respondents. While this study clearly gets to the facts and information components of cholera knowledge, it is sparse on the skills acquisition aspects.

## Conclusion

Cholera is endemic in Ghana with over 7 million people living in cholera hotspots. James Town, a poor urban community, which experiences periodic and seasonal outbreaks, was selected for this study. In this paper, we have shown that basic knowledge of cholera risk factors, although ubiquitous, is differentiated by sex and educational attainment. An enhancement in health literacy (disease knowledge) on cholera environmental risk factors, especially on the medium of coastal water and overcrowded spaces, may improve the well-being of the people by reducing the possibility of cholera infections. This study has evidently illustrated the positive association between better knowledge on cholera risk factors and healthier behavior, specifically better food safety and personal hygiene practices. The study also found that better food safety and personal hygiene practice were significantly associated with lower probability of being infected with cholera. A conscious effort in encouraging consistency in the translation of disease knowledge into healthier practices across age, sex, and educational background may be a way of reducing the impact of cholera in this endemic area. Additionally, this study could guide health policymakers and key stakeholders to invest in health literacy-related campaigns to enhance knowledge, attitude, and practices not only towards cholera but also on all foodborne diseases.

## Abbreviations

ANOVA: Analysis of variance
WHO: World Health Organization
